# High-Risk Pulmonary Embolism: A Second Opportunity for Thrombolysis

**DOI:** 10.7759/cureus.107468

**Published:** 2026-04-21

**Authors:** Joana Luís, Ana M de Oliveira, André C Oliveira, Margarida Vera-Cruz, João Gonçalves Pereira

**Affiliations:** 1 Intensive Care Unit, Unidade Local de Saúde Estuário do Tejo, Vila Franca de Xira, PRT; 2 Intensive Care Unit, Hospital CUF Tejo, Lisbon, PRT

**Keywords:** high-risk pulmonary embolism, obstructive shock, repeated thrombolysis, reperfusion strategies, rescue therapy

## Abstract

Pulmonary embolism (PE) remains a major cause of cardiovascular mortality. High-risk PE is characterized by acute cor pulmonale, persistent hypotension, hypoperfusion, obstructive shock, and, in extreme cases, cardiopulmonary arrest. Systemic thrombolysis is recommended for high-risk PE; however, treatment failure may occur in a subset of patients. Rescue options include surgical embolectomy or a second systemic thrombolysis when hemodynamic instability persists. A 71-year-old woman presented to the hospital with sudden-onset dyspnea and severe hypoxemia. Acute cor pulmonale with obstructive shock was diagnosed, and urgent systemic thrombolysis with Alteplase ® (Boehringer Ingelheim, Germany) was started, with initial improvement. Approximately 15 hours later, there was a new significant clinical deterioration, with refractory shock, severe hypoperfusion, and respiratory failure. In the absence of eligibility for surgical or endovascular intervention, a second systemic thrombolysis was performed, resulting in gradual but progressive clinical recovery. She was discharged from the intensive care unit on day 20, eupneic and without supplemental oxygen. Repeat systemic thrombolysis should be considered in high-risk PE that recurs after a first thrombolysis, when reperfusion strategies, such as endovascular therapy or surgery, are not feasible. This case highlights the potential life-saving role of repeated thrombolysis in select critical-care scenarios.

## Introduction

Pulmonary embolism (PE) is a major cause of cardiovascular morbidity and the third leading cause of cardiovascular death worldwide. High-risk PE is associated with significant mortality, which may reach 25-50%, particularly in patients presenting with obstructive shock and acute right ventricular (RV) failure.

RV dysfunction refers to impaired RV performance due to acute pressure overload caused by obstruction of the pulmonary circulation, leading to reduced cardiac output and hemodynamic collapse. Acute cor pulmonale describes acute RV failure secondary to a sudden increase in pulmonary vascular resistance, most commonly due to massive PE. Thrombolysis is a pharmacological treatment that promotes clot dissolution and rapid restoration of pulmonary blood flow.

Beyond traditional risk factors, such as malignancy and advanced age, the incidence of PE has increased due to factors including prolonged immobilization, obesity, postoperative states, and long-distance travel [1]. The 2019 European Society of Cardiology (ESC) guidelines recommend risk stratification of acute PE into high-, intermediate-, and low-risk categories based on hemodynamic status and markers of RV dysfunction, with systemic thrombolysis as first-line therapy in high-risk PE (class I recommendation) [2,3].

Although thrombolysis is effective in restoring pulmonary perfusion, treatment failure or recurrent hemodynamic instability may occur in a subset of critically ill patients. In such situations, rescue strategies include surgical embolectomy or percutaneous catheter-directed interventions; however, these options may not be readily available in all centers or may not be feasible in unstable patients. Evidence regarding repeated systemic thrombolysis remains limited to isolated case reports and small case series, and no standardized recommendations currently exist to guide its use.

This case illustrates the clinical decision-making process and successful outcome of repeated systemic thrombolysis in a patient with high-risk pulmonary embolism and severe RV dysfunction who exhibited recurrent hemodynamic deterioration after initial reperfusion therapy. Although similar cases of rescue or repeated thrombolysis have been reported in the literature, evidence remains scarce, and its safety profile is not well-established [4]. This highlights the need for further studies and registries to better define the role of repeated thrombolysis in carefully selected patients.

## Case presentation

A 71-year-old woman presented to the emergency department with sudden-onset dyspnea. Her past medical history included moderate Alzheimer's disease, a depressive disorder, hypertension, and hypothyroidism.

**Figure 1:**
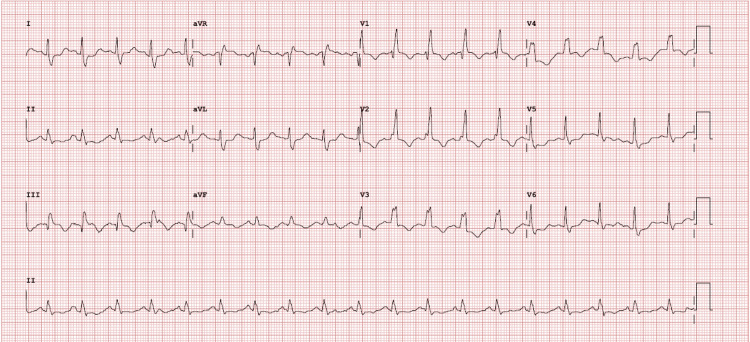
Electrocardiogram Heart rate: 116 bpm; RR interval: 517 ms; PR interval: 116 ms; QRS duration: 129 ms; QT interval: 381 ms; corrected QT (QTc): 530 ms. Electrical axis: P axis: 56°, QRS axis: 59°, T axis: -67°. Standard 12-lead positioning

On arrival to the emergency department, she exhibited severe hypoxemia (SpO₂ of 71% at room air), hypertension (182/103 mmHg), tachycardia (174 bpm), and normal breath sounds. Arterial blood gas analysis showed respiratory alkalosis and partial respiratory failure (Table 1). An electrocardiogram revealed narrow-complex tachycardia (Figure 1).

**Table 1:** Arterial blood gas analysis at the emergency department HCO_3_^-^: bicarbonate; pO_2_: partial pressure of oxygen; pCO_2_: partial pressure of carbon dioxide

Arterial blood gas	Results, units	Normal values
pH	7.43	7.35-7.45
pCO_2_	23.7 mmHg	35-45
pO_2_	43.7 mmHg	70-100
HCO_3_-	15.4 mmol/L	21-26
Lactate	5.4 mmol/L	< 1.8

Her clinical condition deteriorated rapidly, with progressive respiratory distress, altered mental status, hypotension, tachycardia, and signs of peripheral hypoperfusion (mottling score: 4, capillary refill time: >5 seconds) [5,6]. A transthoracic echocardiogram showed significant RV enlargement (right ventricle/left ventricle ratio: >0.9), D-shaped septum, severe tricuspid regurgitation, and estimated pulmonary artery systolic pressure of 80 mmHg, all consistent with acute severe pulmonary hypertension. The Pulmonary Embolism Severity Index (PESI) score was 221 points [7], and she was classified as high-risk PE (30-day mortality: 10-24.5%).

Differential diagnosis at presentation included acute coronary syndrome, severe pneumonia, and acute respiratory distress syndrome, given the presence of acute respiratory failure and hemodynamic instability. These conditions were initially considered and subsequently excluded based on clinical presentation, imaging findings, and laboratory results consistent with pulmonary embolism.

**Figure 2:**
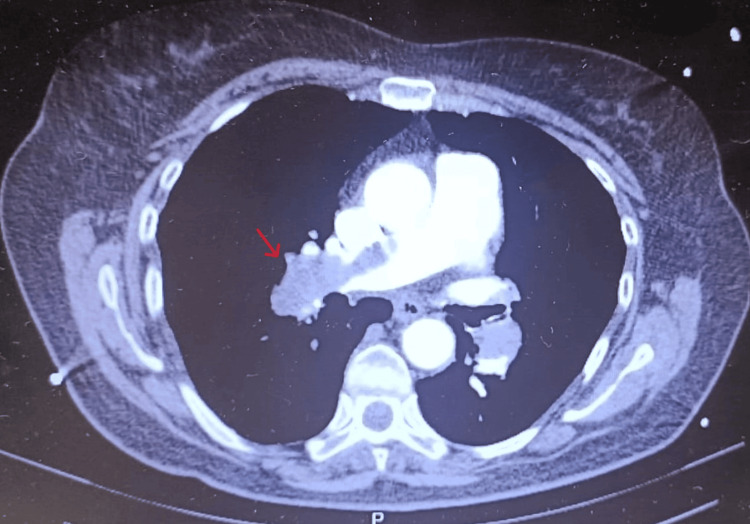
Computed tomography pulmonary angiography (CTPA) demonstrating extensive filling defects in the right main pulmonary artery consistent with acute pulmonary embolism

**Figure 3:**
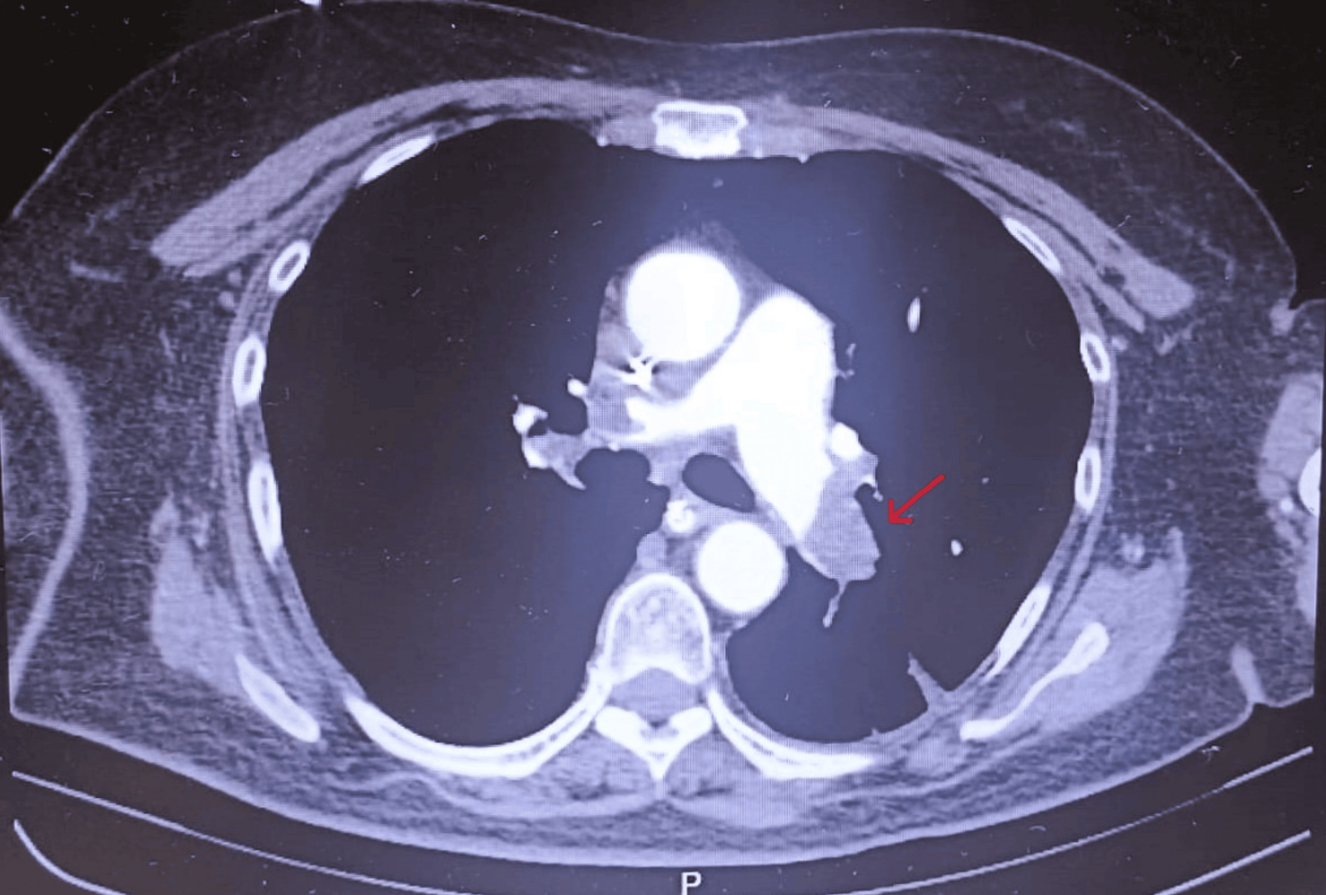
CTPA showing significant filling defects in the left pulmonary artery and segmental branches

Systemic thrombolysis with recombinant tissue plasminogen activator (rt-PA) Alteplase ® (Boehringer Ingelheim, Germany) (100 mg over two hours - 50 mg in bolus over 15 minutes and 50 mg in perfusion) was started, resulting in normalization of the hemodynamic profile and progressive clinical improvement. Computed tomography pulmonary angiography (CTPA) at presentation demonstrated extensive bilateral filling defects involving the main pulmonary arteries with near complete occlusion of segmental branches, associated with RV dilatation and interventricular septal flattening, consistent with acute right heart strain (Figures 2-3). Meanwhile, the results of the clinical analyses also became available, showing an increase in D-dimers (Table 2).

**Table 2:** Clinical test results

Blood analysis	Results, units	Normal values
Hemoglobin	14.2 g/dL	12.0-15.0
Hct (hematocrit)	44%	36-46
Platelets	295,000/uL	150-400
D-dimer	5493 ng/mL	<500
Fibrinogen	390 mg/dL	200-400
Creatinine	1.04 mg/dL	0.55-1.02
Urea	36 mg/dL	19-49
NT-proBNP	9481 pg/mL	< 125
Troponin	99 pg/mL	< 39
C-reactive protein	5.0 mg/dL	< 0.34

The patient showed progressive improvement after admission to the ICU, with recovery of neurological status (conscious, calm, and cooperative). She remained on non-invasive ventilatory support, with stable blood gas values and a lower need for oxygen. From a cardiovascular standpoint, the patient is hemodynamically stable, with no need for vasopressors.

However, approximately 15 hours after the thrombolysis, sudden clinical worsening was noted, with marked dyspnea and hypotension (70/40 mmHg). Her SpO₂ dropped to 60-70% despite high-concentration supplemental oxygen, requiring endotracheal intubation and invasive mechanical ventilation. Despite vasopressor support, she evolved into refractory shock with marked hypoperfusion and persistent hypoxemia. An echocardiogram was performed immediately due to suspected recurrent PE. It again revealed an enlarged right ventricle and septal shift toward the left ventricle, along with elevated pulmonary artery pressure (90 mmHg). Due to the clinical instability, she was deemed unsuitable for inter-hospital transfer for endovascular intervention or surgical embolectomy.

**Figure 4:**
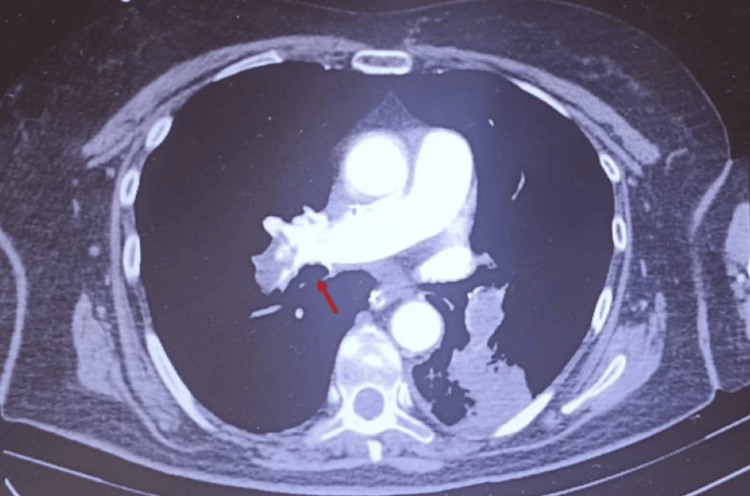
Partial repermeabilization of the right branch of the pulmonary artery after repeated thrombolysis

**Figure 5:**
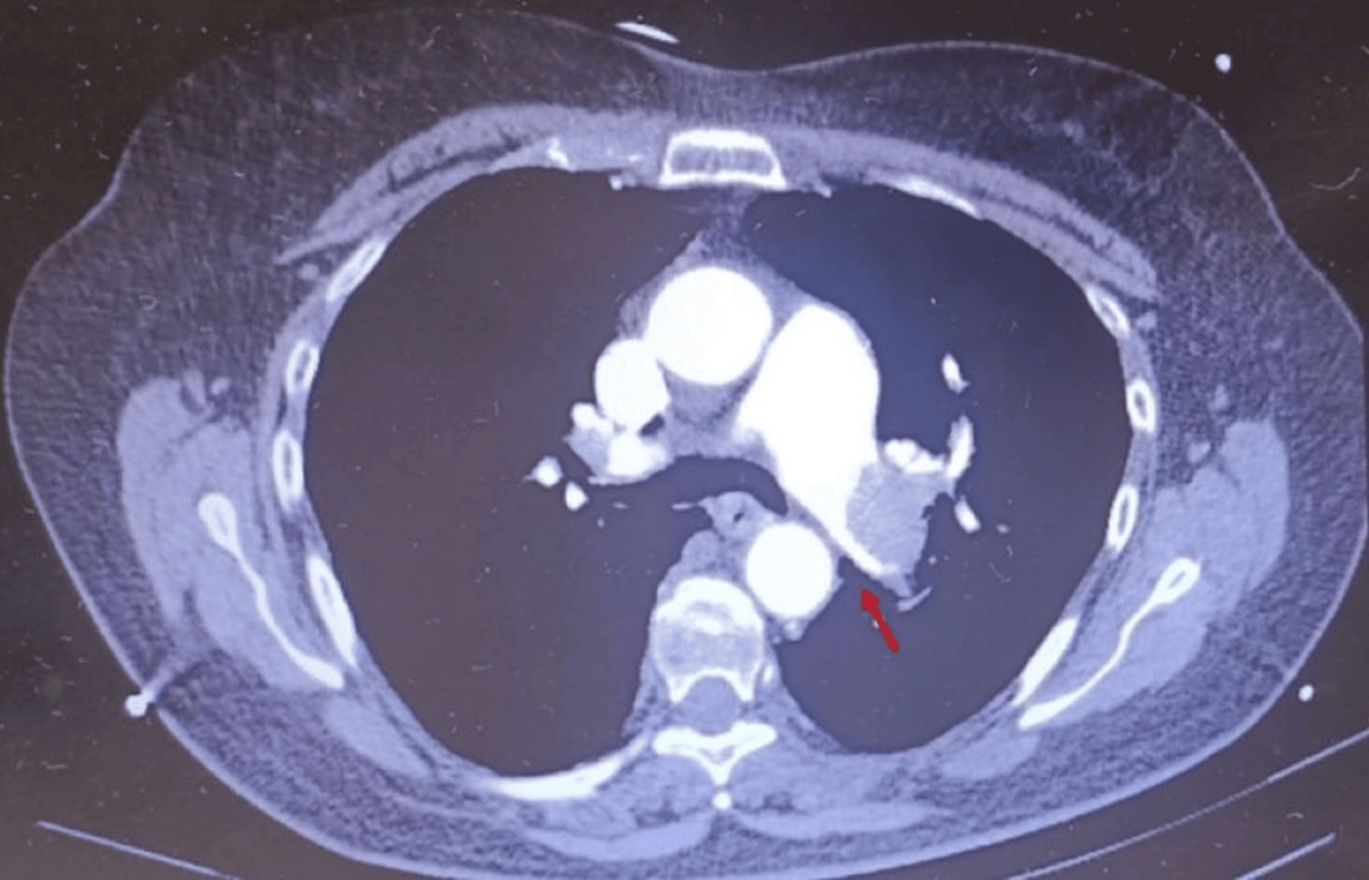
Partial repermeabilization of the left branch of the pulmonary artery after repeated thrombolysis

As a rescue measure, systemic thrombolysis with alteplase (100 mg over two hours) was again administered. No bleeding complications occurred during or after thrombolytic therapy. There was a slow but progressive recovery, with gradual improvement in hemodynamics, oxygenation, and RV function. A thoracic computed tomography was repeated and showed partial repermeabilization of the pulmonary artery (Figures 4-5).

She was weaned from sedation, vasopressors, and invasive mechanical ventilation. Her symptoms improved markedly, and she was discharged from the ICU on day 20, without the need for supplemental oxygen. She was discharged home on day 46, on lifelong hypocoagulation, as shown in the resumen table (Table 3).

**Table 3:** Hemodynamic and clinical timeline

Time point	Hemodynamic status	Lactate	RV function	Vasopressors	Intervention	Outcome
Admission	Shock	↑	RV dilation	NA start	Support	Emergency room
Post 1st thrombolysis	Improved	↑	Partial recovery	↓	Improvement	ICU - stable
15h later	Recurrent shock	↑	Worsened	↑	ICU escalation	Deterioration
Post 2nd thrombolysis	Stable	↓	Improved	↓	Recovery	ICU discharge - 46 days

All clinical scoring systems used in this manuscript (PESI score, mottling score, and capillary refill time) are publicly available and free for clinical and academic use.

## Discussion

We present a case of acute PE recurring after initial thrombolysis. As endovascular intervention or surgery was deemed non-feasible, she was started on a second thrombolysis with significant improvement.

The clinical manifestations of acute PE vary widely, ranging from mild dyspnea to pulseless electrical activity. To guide management, PE is generally categorized into high-risk (massive), intermediate-risk (submassive), and low-risk groups. Risk stratification tools, including the PISI score [7], are commonly used to assess the likelihood of complications and related mortality [4]. High-risk/massive PE represents a cardiovascular emergency with significant mortality. It is defined as sustained hypotension (systolic blood pressure <90 mmHg) lasting more than 15 minutes due to acute PE, the need for inotropic support, or the presence of signs of shock [8]. The ESC guidelines recommend systemic thrombolysis for high-risk acute PE [3], as it remains the primary treatment for patients who are hemodynamically unstable. However, treatment failure occurs in a subset of patients (about 8%), requiring individualized therapeutic rescue strategies [9].

For patients who remain hemodynamically unstable despite thrombolysis, therapeutic options include two main approaches: rescue surgical embolectomy or administering a second thrombolytic treatment [10,11]. Catheter-directed endovascular therapy has gained interest as a potential alternative strategy [12]. Table 4 presents the different case reports with repeated thrombolytic treatment and their outcomes.

**Table 4:** Different case reports with repeated thrombolytic treatment and their outcomes

Author (Year)	Clinical scenario	Indications for repeat thrombolysis	Thrombolytic regimen	Bleeding complications	Outcome
Argüder et al. (2019) [4] - Case 1	56 years old - Intermediate-risk PE	After 5 days, a new, high-risk pulmonary embolism	Alteplase 100 mg in 2 hours	No major or minor bleeding	Survival - discharged home
Argüder et al. (2019) [4] - Case 2	23 years old - High-risk PE	After 5 days, re-embolism suspected	Alteplase 50 mg in 1 hour	No visible bleeding, but Hb 5.4 g/dL	Death - Uncompensated metabolic acidosis
Bigdelu et al. (2023) [9]	45 years old - High-risk PE	Persistent hypotension after initial thrombolysis	Alteplase 100 mg in 2 hours	No major or minor bleeding	Survival - discharged home
Poor et al. (2018) [11]- Case 1	26 years old - High-risk PE with obstructive shock	Persistent pulmonary embolism after first thrombolysis	Alteplase 10 mg bolus + 90 mg infusion; 3 more doses of 50 mg in a 36‑hour period	Hemoperitoneum - 10 units packed red blood cells	Death 1 week after septic shock
Poor et al. (2018) [11] - Case 2	46 years old - High-risk PE with RV failure	Persistent pulmonary embolism after the first thrombolysis	Alteplase 10 mg bolus + 90 mg infusion; 3 more doses of 50 mg in a 36‑hour period	Melena and anemia - 2 units of packed red blood cells	Survival - discharged home
Current case	71 years old - High-risk PE with recurrent hemodynamic instability	Recurrent shock after initial thrombolysis	Alteplase 10mg bolus + 90mg perfusion	No bleeding complications	Survival with recovery

Surgical embolectomy is associated with excellent outcomes when available and feasible. Endovascular direct intervention also provides an alternative mechanical approach to clot reduction and reperfusion. However, both require specialized expertise and may be contraindicated in unstable patients. Repeat thrombolysis remains controversial but is supported by case series and observational data, although increased bleeding risks, especially intracranial hemorrhage (1.7% of patients), have been reported [3].

It seems particularly valuable when the initial thrombolytic response is incomplete or transient; hemodynamic instability persists, or reperfusion strategies are unavailable or contraindicated. In a relatively old case series [8], a second thrombolysis was a safe strategy, but with a high risk of mortality or recurrent PE.

In the present case, recurrent deterioration occurred 15 hours after initial thrombolysis, a period consistent with known thrombolytic failure patterns. Possible reasons for the initial thrombolysis failure in this case include a high thrombotic burden with extensive bilateral pulmonary artery obstruction, which may have limited effective reperfusion, as well as persistent right ventricular dysfunction, leading to ongoing hemodynamic compromise, despite partial clot lysis. In addition, recurrent embolization or an underlying prothrombotic state cannot be excluded.

Alternative reperfusion strategies for high-risk PE include catheter-directed thrombolysis and surgical embolectomy. Catheter-based approaches offer the advantage of lower systemic bleeding risk and targeted thrombus reduction but require specialized expertise and availability. Surgical embolectomy provides definitive clot removal but is associated with a higher procedural risk and is not always feasible in unstable patients.

In the present case, the patient developed persistent obstructive physiology with recurrent hemodynamic collapse. Alternative reperfusion strategies were not immediately available, which supported the decision to perform repeated systemic thrombolysis as a rescue approach. This decision was further supported by the absence of bleeding complications following the initial thrombolytic therapy. The intervention resulted in progressive hemodynamic and respiratory improvement, ultimately leading to complete recovery.

This case reinforces the potential life-saving role of a second systemic thrombolysis in carefully selected patients and highlights the importance of dynamic and multidisciplinary decision-making in high-risk PE.

## Conclusions

The management of high-risk PE remains a major challenge in intensive care medicine due to the high mortality associated with obstructive shock and acute RV failure. While systemic thrombolysis is the recommended first-line therapy, treatment failure or recurrent hemodynamic instability may occur, particularly in critically ill patients or in centers where alternative reperfusion strategies are not readily available. This case suggests that repeated systemic thrombolysis may represent a possible rescue strategy in selected patients with high-risk PE and recurrent hemodynamic deterioration after initial thrombolytic therapy, particularly when other reperfusion options are not feasible. However, this observation is based on a single patient and should therefore be interpreted as hypothesis-generating rather than definitive evidence of efficacy.

The absence of a control group, the potential for selection bias, and the lack of systematic quantitative outcome comparisons (including standardized bleeding rates and longitudinal RV functional assessment) limit the generalizability of the findings. Nevertheless, in this case, clinical recovery without bleeding complications supports the potential feasibility of this approach in highly selected patients. Early recognition of deterioration and prompt intervention remain critical determinants of outcome. Further multicenter studies and prospective registries are required to better define safety, efficacy, and selection criteria for repeated thrombolysis in this rare clinical scenario.
